# Merging cross-border flow optimization techniques for performance maximization

**DOI:** 10.12688/openreseurope.15808.1

**Published:** 2023-09-26

**Authors:** Anastasis Tzoumpas, Alvaro Nofuentes, Catherine Winning, Mark Norton, Zhehan Zhao, Magda Zafeiropoulou, Athanasios Bachoumis, Dusan Vlaisavljevic, Branko Lekovic

**Affiliations:** 1 UBITECH ENERGY, Brussels, Belgium; 2 ETRA I+D, Valencia, Spain; 3 SMART WIRES, Dublin, Ireland; 4 EKC, Belgrade, Serbia

**Keywords:** FARCROSS, TRINITY, SmartValve, T-SENTINEL, cross-border flow optimization techniques, Remedial Action Optimization, Modular Power Flow Controller, Static Synchronous Series Compensator

## Abstract

Both FARCROSS and TRINITY EU research projects aim to increase cross-border electricity flow and regional cooperation. The integration of SmartValve and T-SENTINEL systems offers benefits such as enhancing grid security and reliability, managing thermal constraints, and maximizing utilization of existing infrastructure. The combined system can achieve a more efficient and less costly coordinated network security process, increase cross-border capacities, and promote regional electricity market integration, benefiting the local communities with significant CO2 emissions avoidance and reduced electricity prices. Overall, the integration of SmartValve and T-SENTINEL can provide significant improvements in flexibility, making cross-border connections more robust and adaptive to the evolution of the electrical power industry.

## Plain language summary

The rationale of this paper is to give insights about the way the combination of cross-border flow optimization techniques from two different H2020 projects can lead to a maximization of overall transmission system performance. Specifically, both FARCROSS and TRINITY, strive to address the above-mentioned challenges, especially in the broader area of Southeast Europe, and to achieve the EU goals in energy regarding the establishment of a geographically large market by initially improving its cross-border electricity interconnections.

## Introduction

The commission regulation (EU) 2015/1222 of 24 July 2015 (and modified in March 2021) establishing a guideline on capacity allocation and congestion management (“CACM regulation”) sets out rules to ensure optimal use of the transmission infrastructure, operational security and optimizes the calculation and allocation of cross-zonal capacity. These factors are of crucial importance in order to improve the security of transmission system operation, as well as to foster better coupling of markets. The EU strives to achieve market integration and price convergence within its region; the term is well-known as market coupling. Electricity markets used to be localized and isolated, with demand being covered internally and a low amount of cross-border energy flows. The transition to integrated markets implies that high amounts of electricity are traded among the EU member countries, increasing the security of supply, while at the same time the cost of supply is being decreased. The ultimate measure of market integration is the degree of cross-border price harmonization. However, this is still far from achieved. As the Market Monitoring report in 2019 of the European Union Agency for Cooperation Energy Regulators (ACER) states, on about 50% of all European borders, the absolute cross-border differences in prices are higher than 5%, while on about 10 borders the absolute price difference exceeds 10%.

In the Clean Energy of all Europeans Package (CEP), the lack of sufficient cross-zonal capacity is considered as one of the main barriers to the integration of electricity markets. Hence, installing more cross-border capacity is one of the main ambitions. European Network of Transmission System Operators for Electricity (ENTSO-e) in the “Completing the map Power system needs in 2030 and 2040” report has identified that by 2040, 93 GW of additional cross-border capacity in Europe is required. In addition, other solutions such as storage, hybrid offshore infrastructure, smart grids, Power-to-X conversion technologies should be integrated in order to facilitate a Pan-European efficient electricity market. Besides the expansion in terms of cross-border capacity through traditional grid upgrades, increase in cross-border efficiency will also enhance market coupling. By achieving market coupling through efficient utilization of the existing infrastructure, while ensuring power system security, there are significant benefits from an environmental perspective, as well as an opportunity to minimize the total costs for the end-consumers.

ACER states that the development of European rules for the calculation and allocation of cross-zonal capacities on electricity interconnectors is an integral step in order to increase market efficiency. Therefore, as of January 1st, 2020, Article 16.8 EU 2019/943 was introduced, implying that TSOs shall make available a minimum binding level of capacity equal to 70% for cross-zonal trade. According to this rule, the capacity made available to market parties per cross-border connection must be higher than 70% of the technical capacity, after controlling for a reliability margin to deal with loop flows and emergency conditions. For cross – border connections using a coordinated net transmission capacity approach, the minimum capacity must account for 70% of the transmission capacity, respecting operational security limits after deduction of contingencies. For cross – border connections using a flow-based approach, the minimum capacity shall be a margin set in the capacity calculation process as available for flows induced by cross-zonal exchange. The margin shall be 70% of the capacity respecting operational security limits of internal and cross-zonal critical network elements, taking into account potential contingencies. Nevertheless, based on the ACER report about the minimum capacity margin available for cross-zonal trade (MACZT) in the first semester of 2020, even though on Direct Current (DC) borders the 70% target was met most of the time with a few notable exceptions, on Alternating Current (AC) borders, there is a very diverse picture with significant room for improvement to meet the 70% target for most regions and borders. Especially in the Southeast Europe region, the MACZT, in almost all of the interconnections, was not achieved even for 10% of the total hours.

Due to the relevance of this topic, research and innovation in this field is being financed by different governmental bodies, with Horizon2020 being one of the most relevant innovation programmes in Europe. The rationale of this paper is to give insights about the way the combination of cross-border flow optimization techniques from two different H2020 projects can lead to a maximization of overall transmission system performance. Specifically, those two EU-funded projects, namely FARCROSS and TRINITY, strive to address the above-mentioned challenges, especially in the broader area of Southeast Europe, and to achieve the EU goals in energy regarding the establishment of a geographically large market by initially improving its cross-border electricity interconnections.

## Software solution: T-SENTINEL TOOLSET

Coordinated Security Analysis (CSA) is one of the main tasks conducted by Regional Security Centers (RSCs). During CSA, overloads can be detected on some monitored Critical Network Element & Contingencies (CNECs). In order to maintain the loading of CNEC within operational security limits, RSCs can implement a relevant set of Remedial Actions, including costly ones (e.g. Redispatching). After implementing selected costly RA, a Cost Sharing process is conducted. All three layers of CSA: Security monitoring, Remedial Action and Cost sharing are regulated within a comprehensive EU legal framework: SOGL with related CSAM and ROSC Methodologies, and CACM and related Cost Sharing Methodologies.

One of the aims of TRINITY project is to develop novel methodologies and deliver a multi-modular toolset that will enhance the European Transmission System Operators (TSOs) and Regional Security Coordinators business processes.

For this purpose, the power system security and reliability T-SENTINEL toolset, which includes the development of novel algorithms and corresponding IT solutions related to coordinated security assessment functions has been developed. Under T-SENTINEL TOOLSET, security monitoring task is enhanced with two back-to-back modules: Remedial Action Optimization (RAO) and Redispatching Cost Sharing (RDCS).

**Figure 1.**
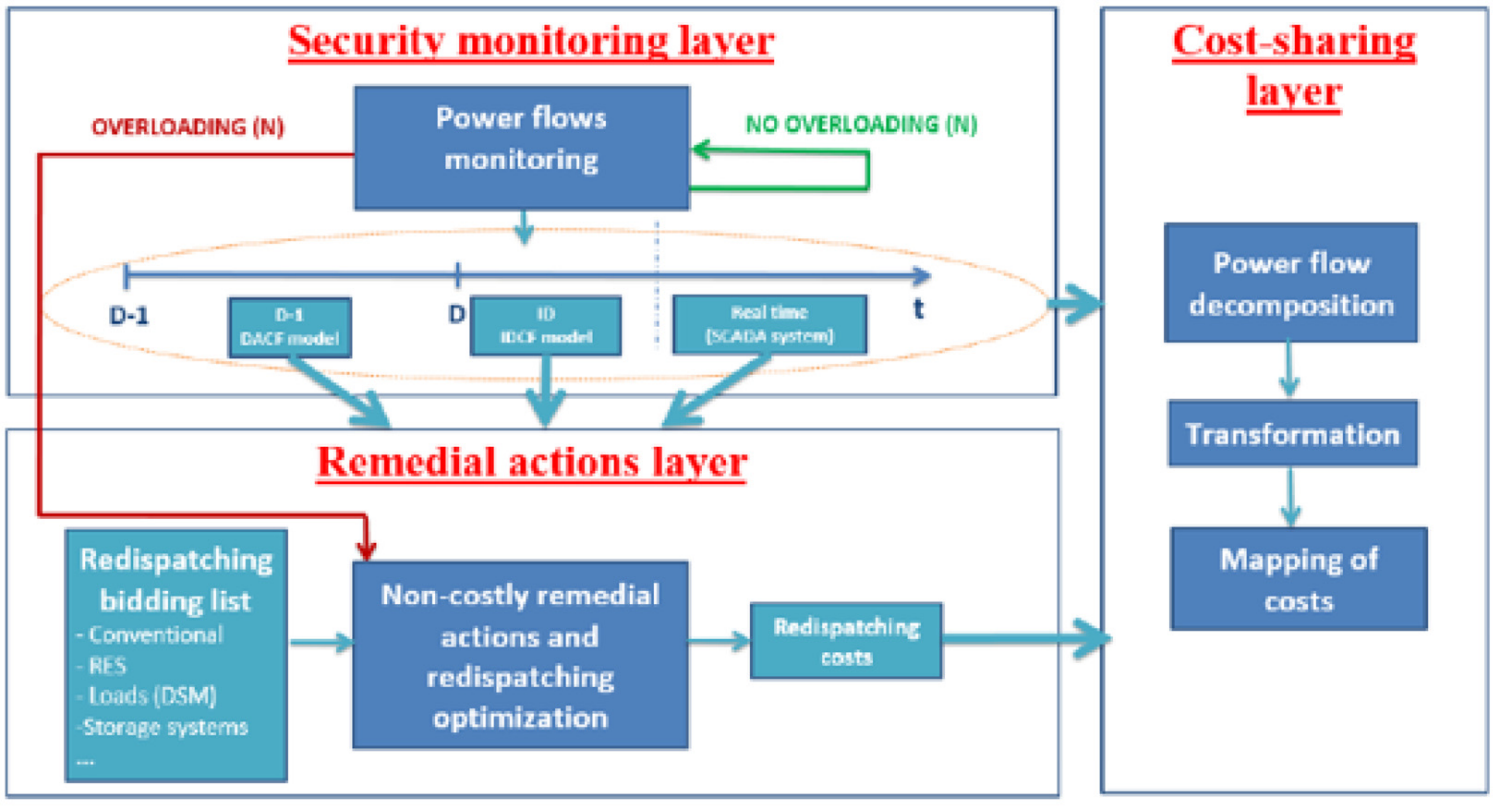
Scheme of T-SENTINEL network security module.

The T-SENTINEL network security module ( Figure 1) is expected to deliver advancements in the context of (1) improved efficiency of solving network congestions through co-optimization of non-costly and costly redispatching actions and (2) better sharing of the overall costs of redispatching.

The T-SENTINEL Remedial Action Optimization module (RAO module) is responsible for performing day ahead and intraday power system static security checks and optimization of coordinated multi-lateral remedial actions when potential overloads are detected.

The T-SENTINEL Redispatching Cost Sharing module (RDCS module) contains fully implemented mechanisms for fair distribution of redispatching actions costs among the transmission system operators. It encompasses three different processes, which are power flow decomposition, transformation and mapping.

The T-SENTINEL Remedial Action Optimization (RAO) module aims to choose the least expensive Remedial Action solution considering costly redispatching actions, but also non-costly actions such as adjusting active power flow through HVDCs and impacting power flow by tap changes of the PSTs.

The algorithm of proposed TRINITY solution for the RAO layer consists of the following steps:

1. Generating and preparing Input files for Remedial Action Optimisation.
  - The goal of the preparation submodule is to prepare the main input files for RAO: ORCA and RAF files. ORCA file represents enhanced CNEC list with relevant sensitivity factors (nPTDF – nodal power transfer distribution factors, PSDF – phase shift transformers distribution factors and DCDF – DC elements distribution factors). RAF file represents a predefined file in which RA candidates for optimisation are described. As a first step of the preparation phase, the RAF file is updated with the current PST/HVDC position/flows, and then together with relevant CGM, CNEC and GSK files, it is used to generate ORCA file.
2. Remedial Action Optimisation, is performed based on the following principles:
  - Minimisation of incurred costs.
  - Remedial Actions (RAs) will be balanced, meaning that the cumulated fed-in active power into the electrical grid will not change.
  - RAO will not lead to additional violations of operational security limits.
  - The non-costly topological actions will be considered first, as the initial step of the process, based on the principle of selecting actions which generate the maximum deloading factor of overloaded elements. Hence, the costly RAO is triggered in sequential manner if non-costly solution cannot be found.
3. Verification:
  - Verification of DC-based optimisation results is performed. By using relevant common grid model (CGM) and RAO results (selected preventive and curative actions), full AC load flow is conducted for N state and each relevant N-1 state, so that the proposed remedial actions are checked under AC load flow simulation.
4. Reporting:
  - After the optimisation phase, RAO results with all relevant data are sent to the T-COORDINATION platform for further coordination. T-COORDINATION platform is also being developed under TRINITY project, as a tool which will enable communication and coordination between regional TSOs and their RSC.

Full mathematical formulation of the applied algorithm can be found in Deliverable 4.2 of TRINITY ^1^.

## Hardware solution: MPFC

**Figure 2.**
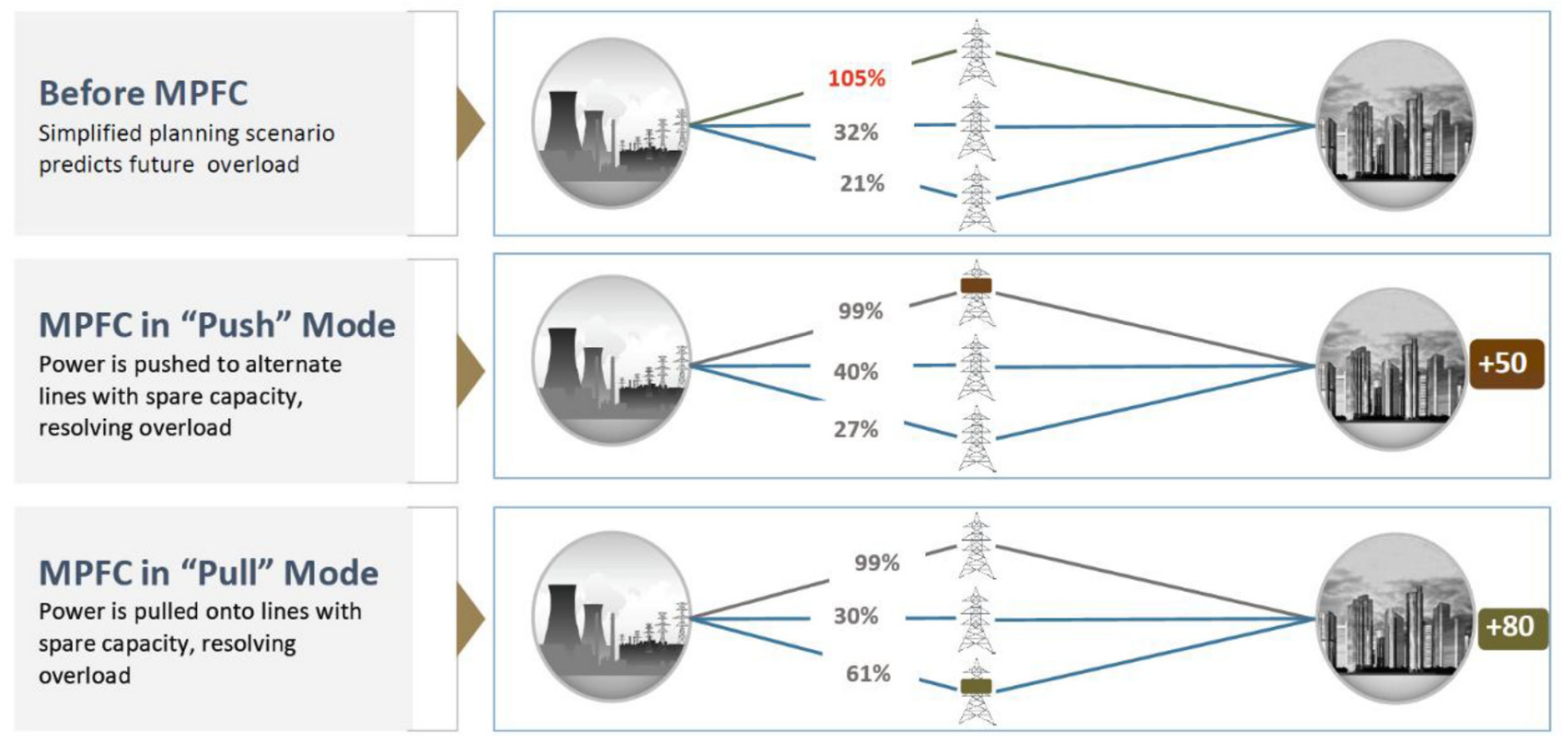
The use of MPFC to increase power flows.

MPFC Technology is able to deliver additional capacity through the redistribution of power flows. Typically, networks have several routes for power flow to travel, these routes have different impedances resulting in an unequal distribution of power flows. One route becomes overloaded first, limiting the utilisation of the network to protect the respective devices. Power Flow Control is able to redistribute the power flows allowing greater use of existing network capacity. An example of this is illustrated in the Figure 2.

The proposed modular solution allows much smaller levels of impedance to be strategically deployed to maximize overall utilization than is practical with other technologies. In this way, it is possible to alleviate strains inflicted on transmission lines occurring at border regions. In addition, it can mitigate restrictions in the network that prevent power flowing to the cross – border connections, also limiting cross border capacity. Power Flow control technology differs from many other technologies as it can act both in a capacitive or inductive mode to push power away from an overload or pull it towards an under loaded circuit.

For the FARCROSS demonstration project a Smart Wires solution including two SmartValves per phase is envisaged ^2^. There are several bespoke aspects of SmartValve technology that must be considered as part of the process to assess suitable deployment locations.

The SmartValve is based on the Static Synchronous Series Compensator (SSSC) technology. This insulated-gate bipolar transistor (’IGBT’) based technology makes it ideal for deploying in places where there are concerns on system interaction, such as the appearance of sub-synchronous resonances, or a requirement to rapidly respond to changing system conditions.

To accommodate changing system conditions, the mSSSC technology can operate in a number of different control modes, rapidly move between them and change between capacitive and inductive injection mode. This flexibility in its operating principles and control capabilities offers a number of benefits and is particularly helpful for the management of power flows driven by intermittent renewable energy sources.

The modular nature of the solution allows adjustment of transmission line reactance at a granular level in real-time to control network power flows. This approach allows a small number of devices to be installed on several lines; small amounts of impedance on several circuits can typically be very effective at increasing overall power flows whilst limiting the amount of required impedance. Whilst not the focus of this initial deployment, the solution can be built upon and expanded in a coordinated way at a future stage to enhance the cross – border impact initially delivered.

Furthermore, its modular nature allows a similar future solution to be easily scaled or reduced in size as transmission needs change. This allows the project to investigate areas where needs are only temporary, with greater reinforcement able to be delivered at a later date. Once the final solution has been delivered the solution could be redeployed to resolve a different issue. Also, the device can be used in areas where the network requirements are likely to be increased at a later date, for example due to an increase in local renewable connections. The solution can then be upgraded with additional modules, once the exact future need is known.

**Figure 3.**
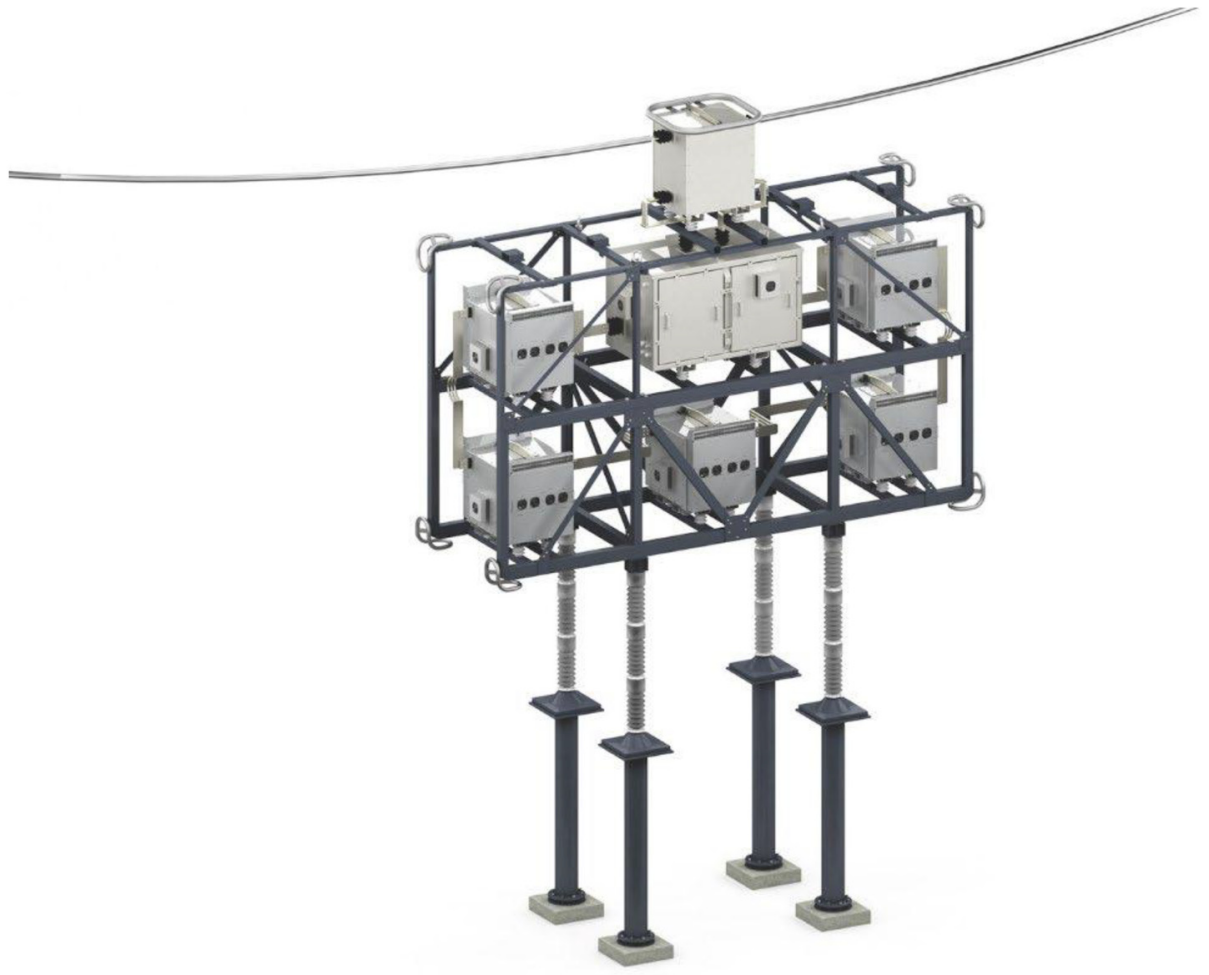
Layout of ground based 1–1800 SmartValves.

**Figure 4.**
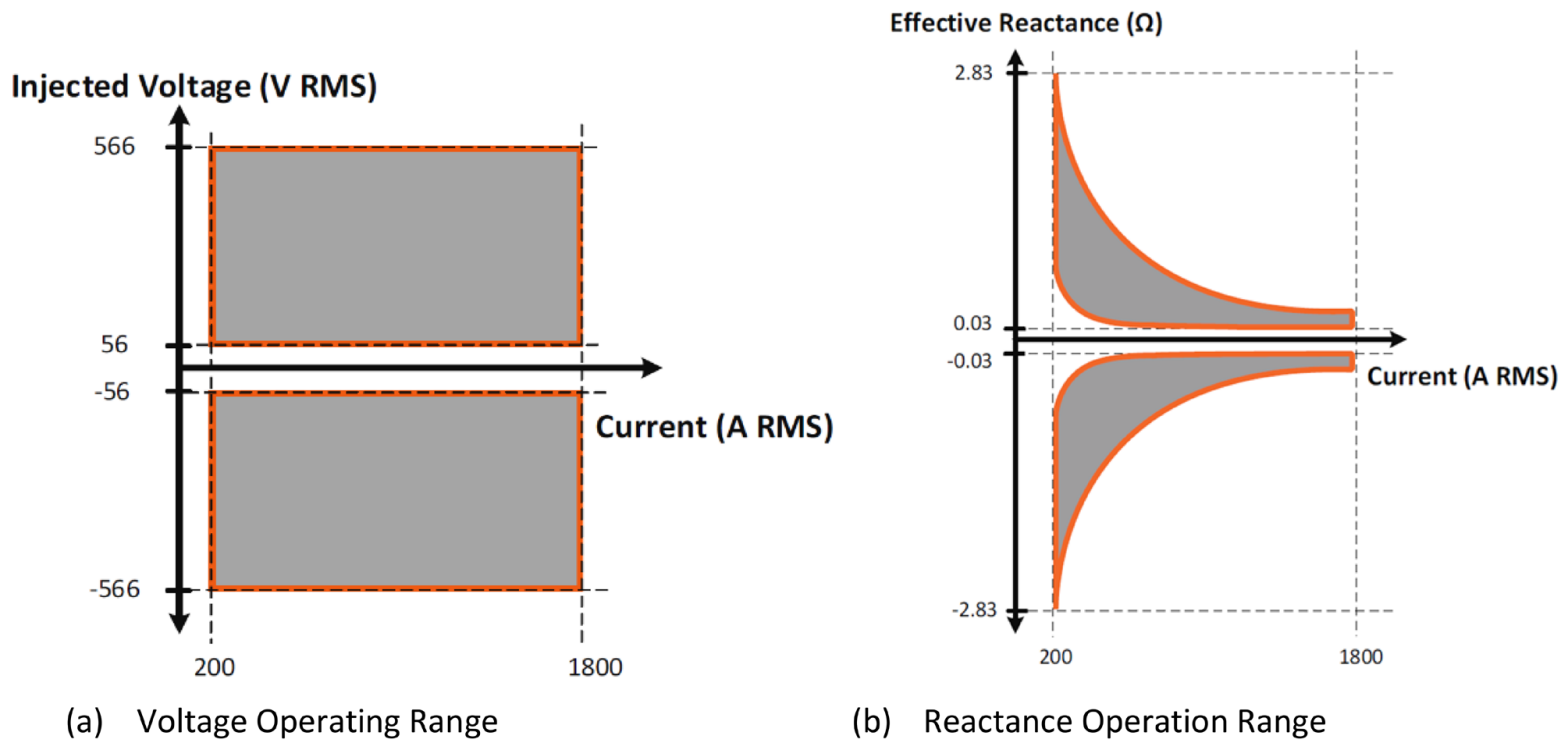
SmartValve 1–1800 Operating Range.

For the FARCROSS project, the ground based 1-1800 SmartValve, as shown in Figure 3, is expected to be supplied. This device can accommodate up to 1800 A RMS, and can be installed at any voltage level. There is a minimum current requirement of 100 A to enter monitoring mode and 100 A to enter injection mode, so the device is operational between 200A and 1800 A, as shown in Figure 4-(a). When considering deployment these aspects must be included, on lightly loaded lines where the intention is pull additional power on to the line. In such a case, it must be assured there is sufficient current to allow the device to operate, or that power can be diverted (maybe by another SmartValve deployment on an adjacent circuit) to make this possible.

Conversely, when the SmartValve is used to push power away from overloads, this should not exceed a continuous current of 1800 A. It is expected that through the consideration of its built – in push and pull functionality, there will be potential of deployments that allow operation within this envelope.

The SmartValve’s capability to inject up to a fixed maximum reactive voltage, results in a non- linear operating range, as seen in the Figure 4-(b). The effective impedance delivered is dependent on the line current where the device is installed. In Smart Wire’s experience there is generally a smaller level of impedance required when mSSSC technology is deployed on the overloaded circuit that needs to be reduced. However, this can still result in the requirement of more than one devices, due to the lower effective impedance injection from each module at high current. Therefore, a more effective solution can be to use the devices in pull mode, requiring a higher level of impedance, but potentially a smaller number of devices. To optimise the effectiveness of the solution against its cost, this relationship must be taken into account when assessing the network’s needs.

Moreover, the operation of the network is dynamic, with the timing of peak circuit loading varying between circuits. This means that a dispersed solution with a number of units working in unison can be optimal to centralise at one location, providing significant efficiency benefits.

## Complementarity analysis and expected results

The objective of the project is to increase the cross – border electricity flow and regional cooperation. Based on the study of both technologies, the integrated solutions can provide a number of benefits for regional power systems to facilitate local power control and electricity market interaction, as well as to enhance their grid security and reliability.

A study on the SmartValve’s installation indicates that the project effectively reduces the thermal loads on the installed line during normal operation stage and N-1 contingency by redirection of the power flow to under-utilised lines. By optimizing the power flows, the solution enables system operators to manage the thermal constraints and maximize the utilization of the existing infrastructure. Combining the T-SENTINEL and SmartValve Systems, it is expected to leverage the control systems’ flexibility to maximize the effectiveness of the devices. The Remedial Action Optimization allows the utilities to optimise SmartValve System control following the real-time operational violation calculation. As a result, it will significantly enhance the local power system security, stability and reliability.

By implementing the combined system, a more efficient and less costly coordinated network security process will be achieved in the SEE region. In addition, the SmartValve System, in synergy with T-SENTINEL module related to capacity calculation, can enable system operators to increase the cross-border capacities. This would yield to overall increase of regional power exchange, regional electricity market integration and consequently social economic welfare in the region.

The advanced control system of the SmartValve enables power flow management in real-time. Furthermore, it allows system operation to effectively manage the dynamic local renewable generation integration. By removing local grid constraints, SmartValve allows regional renewable energy to be connected and more consistently utilised in the grid, reducing the need for conventional generation. Therefore, the project benefits the local communities with significant CO _2_ emissions avoidance and a reduced price for electricity. It also simultaneously strengthens the reliability and resilience of the system, reducing the required size of system reserves by better management and reach of their use.

The modular nature and small size of SmartValve enables it to be easily scaled, up or down, and to be rapidly deployed or re – deployed. It permits the SmartValve System to perform as an interim solution for outage management, reconductoring deferral, failure recovery and asset management to minimize the investment risk and mitigate the system risks. In this sense, the advanced optimization system of T-SENTINEL allows utilities to optimize the deployment location of SmartValve System to maximize the flexibility of SmartValve system.

Additionally, recent analysis shows that the Smartvalve is able to support voltage regulation and power oscillation damping. This is accomplished by the adjustment of the circuits’ reactance, to compensate the reactive power to support voltage control. Meanwhile, the advanced control system enables SmartValve to respond dynamically in a voltage collapse. Therefore, combined with T-SENTINEL it can provide additional ancillary services to enhance the security of the grid.

## Conclusions

Cross – border power transmission connections in the SEE countries typically suffer from congestion, lack of contingency and market inconsistencies. At the same time, the available Renewable Energy resources aren’t injected to the transmission corridors in a cost – effective and secure way, making the need for transmission asset upgrade vital. In this sense, the operational principles and benefits of two different solutions are presented within the specific scope of work. On a network level, T–SENTINEL remedies grid overloading conditions, through the configuration of optimal economic dispatch commands and extended static security monitoring. On a hardware level, SmartValves are introduced as a means to divert power flows from congested lines, as well as to distribute all generation available in between the transmission lines in a more secure and tolerant way. Consequently, the utilization of both technologies can be expected to offer significant improvements in terms of flexibility, making the cross – border connections more robust and adaptive to the evolution of the electrical power industry.

The above-described qualitative analysis provides excellent overview of potential synergies and expected benefits from the combination of the two products. The further simulations and quantitative analyses can be performed after agreement with the SEE TSOs related to the network models provision. For this, both consortiums will promote the potential of this research looking for the necessary support from external bodies.

## Disclaimer

The views expressed in this article are those of the author(s). Publication in Open Research Europe does not imply endorsement of the European Commission.

## Data Availability

No data are associated with this article.
