# Supplementary material for: Merging cross-border flow optimization techniques for performance maximization
Source: Open Res Eur. 2023 Sep 26;3:161. [Version 1] doi: 10.12688/openreseurope.15808.1 (PMC10665604; doi:10.12688/openreseurope.15808.1)
Supplement: Peer review history

# Reviewer report: Reviewer response for version 1

**DOI:** 10.21956/openreseurope.17078.r36116

**Published:** 2023-11-22

**Authors:** Dimitrios C. Rakopoulos, Maria Fotopoulou

**Affiliations:** 1 Department of Thermal Engineering, School of Mechanical Engineering, Centre for Research and Technology Hellas, Athens, Greece; 2 Centre for Research and Technology Hellas (CERTH), Athens, Greece

The authors tackle a modern topic, which is the cross-border flow optimization. A few minor comments, that could enhance the quality of the manuscript, are presented below:

1. What is the TRL of the presented technologies?
2. The authors are encouraged to include formulas/flowcharts/architectures that showcase the operating principle of the proposed systems.
3. The authors could add KPIs and their target values in order to highlight the impact of the proposed solution.

Taking the aforementioned comments into consideration, the manuscript is approved with minor revisions.

Where applicable, are recommendations and next steps explained clearly for others to follow? (Please consider whether others in the research community would be able to implement guidelines or recommendations and/or constructively engage in the debate)

Yes

Does the article adequately reference differing views and opinions?

Yes

Are all factual statements correct, and are statements and arguments made adequately supported by citations?

Yes

Is the rationale for the Open Letter provided in sufficient detail? (Please consider whether existing challenges in the field are outlined clearly and whether the purpose of the letter is explained)

Yes

Is the Open Letter written in accessible language? (Please consider whether all subject-specific terms, concepts and abbreviations are explained)

Yes

Reviewer Expertise:

sustainable energy systems

We confirm that we have read this submission and believe that we have an appropriate level of expertise to confirm that it is of an acceptable scientific standard.

# Reviewer report: Reviewer response for version 1

**DOI:** 10.21956/openreseurope.17078.r35337

**Published:** 2023-10-12

**Authors:** Venizelos Efthymiou

**Affiliations:** 1 American University of Cyprus, Larnaca, Larnaca, Cyprus

The article "Merging cross-border flow optimization techniques for performance maximization" covers quiet vividly the valuable work done within the projects FARCROSS and TRINITY EU. It addresses profound issues that EU is facing with cross border flow of energy in support of the prevailing market. The presented tools to enhance inter-state trading SmartValve and T-Sentinel were presented with good results. The description and cross reference is clear giving to the reader a complete picture of the achieved results. Future work is clearly spelled out and it is responsive to the current achieved results.

Where applicable, are recommendations and next steps explained clearly for others to follow? (Please consider whether others in the research community would be able to implement guidelines or recommendations and/or constructively engage in the debate)

Yes

Does the article adequately reference differing views and opinions?

Yes

Are all factual statements correct, and are statements and arguments made adequately supported by citations?

Yes

Is the rationale for the Open Letter provided in sufficient detail? (Please consider whether existing challenges in the field are outlined clearly and whether the purpose of the letter is explained)

Yes

Is the Open Letter written in accessible language? (Please consider whether all subject-specific terms, concepts and abbreviations are explained)

Yes

Reviewer Expertise:

Power systems, smart grids, microgrids, protection and control of power systems, storage, PV technologies,

I confirm that I have read this submission and believe that I have an appropriate level of expertise to confirm that it is of an acceptable scientific standard.
